# Transcriptomic analysis of naturally occurring bovine respiratory disease in Australian feedlot cattle identifies bovine viral diarrhoea virus-induced immunosuppression and *Mollicutes* as the dominant drivers of lung inflammation

**DOI:** 10.1186/s13567-026-01813-y

**Published:** 2026-08-18

**Authors:** Gebremeskel Mamu Werid, Farhid Hemmatzadeh, Darren Miller, Milton M. McAllister, Darren J. Trott, Kiro Petrovski

**Affiliations:** 1 Australian Centre for Antimicrobial Resistance Ecology, School of Animal and Veterinary Sciences, Adelaide University, Roseworthy, SA 5371 Australia; 2 Davies Livestock Research Centre, School of Animal and Veterinary Sciences, Adelaide University, Roseworthy, SA 5371 Australia

**Keywords:** RNA-seq, Mollicutes, *Pasteurella multocida*, *Histophilus somni*, WGCNA, bovine respiratory disease, bovine viral diarrhoea virus

## Abstract

Bovine respiratory disease (BRD) is the most economically significant syndrome of feedlot cattle worldwide, yet the relative contributions of individual BRD pathogens to host transcriptional responses remain poorly understood. We performed pathogen-stratified transcriptomic analysis of lung tissue obtained from naturally occurring BRD cases (*n =* 22) and non-BRD controls (*n =* 9) in feedlot cattle. BRD-affected tissue showed significant immune activation with 3148 differentially expressed genes (DEGs; 18.7% of the 16,873 genes tested), of which 70.4% were upregulated. Pathogen-stratified analysis revealed significant heterogeneity in host response (221–2241 DEGs per pathogen). Bovine viral diarrhoea virus (BVDV) was associated with a distinct immunomodulatory signature (79% of genes downregulated), consistent with its immunosuppressive role. Mollicutes showed the strongest transcriptional overlap with the core BRD signature: *Mycoplasmopsis arginini, Metamycoplasma alkalescens* and *Metamycoplasma canadense* shared 53–71% of their differentially expressed genes with the core BRD signature with 100% directional concordance, whereas *Mycoplasmopsis bovis* showed lower overlap (34%) despite near-ubiquitous detection (87.1%). *Mycoplasmopsis arginini* induced the largest transcriptional footprint (2241 DEGs), with 1316 genes (58.7%) overlapping the core BRD signature, representing 41.8% of all BRD DEGs*,* suggesting it may have an important role in BRD pathogenesis. *Pasteurella multocida* and *Histophilus somni* ranked among the top three in the integrated analysis, accounting for 77% of dose-responsive gene associations with load-dependent inflammatory amplification. It is hypothesised that in naturally occurring BRD in feedlots, BVDV was associated with suppressed host immunity; Mollicutes were associated with the core inflammatory transcriptome; and Gram-negative bacteria were associated with amplified host response proportionally to the bacterial load. These findings provide an important rationale for future diagnosis, treatment, prevention and control of BRD in Australian feedlots.

**Supplementary Information:**

The online version contains supplementary material available at 10.1186/s13567-026-01813-y.

## Introduction

Bovine respiratory disease (BRD) represents the most economically significant syndrome affecting feedlot cattle worldwide [1]. Annual losses attributable to BRD in North America alone exceed $3 billion USD, consisting of both direct costs associated with treatment and mortality and indirect costs such as reduced growth performance, lower carcass quality and increased labour [2]. In Australia, BRD has emerged as a significant constraint on feedlot productivity, with increasing recognition of its economic impact on the beef industry [3].

BRD is a multifactorial syndrome arising from interactions between environmental, host and pathogen-specific factors [1, 3]. From a pathogen perspective, BRD is classically described as a polymicrobial disease complex in which primary viral infection predisposes the host to secondary bacterial pneumonia [4, 5]. The primary viral agents associated with BRD include bovine herpesvirus 1 (BoHV-1), bovine viral diarrhoea virus (BVDV), bovine respiratory syncytial virus (BRSV) and parainfluenza 3 virus (PI3V), while the principal bacterial pathogens comprise *Mannheimia haemolytica*, *Pasteurella multocida*, *Histophilus somni* and various* Mollicute* species, of which *Mycoplasmopsis bovis* is generally regarded as the most significant [4–7].

Despite decades of research focused on BRD pathogenesis, significant knowledge gaps persist regarding the role of specific pathogens and their relative contributions in a polymicrobial setting [8, 9]. Additionally, while commercial vaccines targeting some viral (BoHV-1) and bacterial pathogens (*M. haemolytica*) have been successfully integrated into BRD management programmes, adequate control of BRD is still largely based on metaphylactic antimicrobial protocols which show inconsistent efficacy, partially due to emerging drug resistance [10, 11]. We recently applied meta-transcriptome and metagenome profiling to the same bovine lung tissue samples and demonstrated that the lung virome is dominated by bacteriophages [12]. In the same study, BVDV was identified as the most abundant BRD-associated virus in samples from cattle with BRD compared to non-BRD controls, and metagenome analysis revealed that *P. multocida* and several Mollicutes, including *Mycoplasmopsis arginini* and *M. bovis,* were significantly more abundant in cattle with BRD compared to non-BRD controls [12]. These findings underscore the polymicrobial nature of BRD and highlight the need for integrative analytical approaches that account for multiple co-infecting agents. A comprehensive, systems-level understanding of how specific BRD pathogens shape the host response could lead to more effective treatment and prevention strategies.

Transcriptomic approaches are powerful tools for dissecting host–pathogen interactions by quantifying genome-wide gene expression changes in response to infection. Previous BRD transcriptomic studies have primarily employed experimental challenge models with single pathogens, providing valuable but potentially incomplete pictures of the natural syndrome [13, 14]. Blood-based transcriptomics have identified systemic immune signatures but cannot capture local pulmonary responses where disease pathology occurs [14–17]. Transcriptomic studies of naturally occurring BRD at the site of primary pathology within infected lung tissue remain limited, and systematic comparisons of transcriptional responses that account for multiple co-infecting pathogens within a single population have not been reported. Furthermore, most studies have not considered the vaccination status of study animals, making it difficult to distinguish intrinsic pathogen contributions from vaccine-modified responses. To the best of our knowledge, no previous study has performed pathogen-stratified transcriptomic analysis directly in bovine lung tissue from naturally occurring BRD cases, representing a critical gap in our understanding of the molecular pathogenesis of this economically important disease complex.

In the present study, we hypothesised that bovine lung tissue obtained from naturally occurring BRD cases (in comparison to healthy control lungs [control]) would show characteristic transcriptional signatures reflecting both conserved host responses and pathogen-specific modulation. Furthermore, systematic analysis of the bovine lung transcriptome in parallel with the BRD microbiome/virome may quantitatively rank the contributions of each disease-causing agent to overall BRD pathogenesis. Thus, the specific objectives of the current study were to characterise the global transcriptional landscape of BRD through differential gene expression and functional enrichment analysis; identify co-expressed gene modules associated with BRD status; define pathogen-specific transcriptional signatures; quantify pathogen contributions to the core BRD phenotype; and assess dose–response relationships between pathogen load and gene expression.

## Materials and methods

### Study design and sample collection

Using a cross-sectional study design, lung tissue samples were collected from Angus steer cattle at a commercial Australian feedlot over a 2-year period, from March 2024 to May 2025. All samples were obtained during the peak BRD season (March–May), and both cattle with BRD and cattle without BRD (BRD and non-BRD cattle groups, respectively) were represented in each collection year. All cattle were vaccinated at induction against BoHV-1 (Rhinogard vaccine; Zoetis Ltd., Parsippany–Troy Hills, NJ, USA) and *M. haemolytica* (Bovilis MH vaccine; Coopers Animal Health, Macquarie Park NSW, Australia). Cattle were assigned to either the BRD group or the non-BRD control group based on antemortem clinical records and post-mortem pathological findings. Cattle showing clinical signs consistent with BRD, including nasal and/or ocular discharge, coughing, abnormal or laboured respiration, drooping ears, and elevated temperature, were identified during routine pen-rider health checks. These animals were pulled for treatment and administered antimicrobial therapy (tulathromycin, tilmicosin or oxytetracycline). Lung tissue was collected from 23 BRD-affected cattle that either died or were euthanised, comprising 22 treatment-refractory cases and one animal that died before antimicrobial therapy could be initiated. Lung tissue samples from non-BRD cattle (*n =* 9) were collected from cattle that showed no respiratory signs during their feedlot stay and did not receive BRD-specific treatment. These animals either died or were euthanised due to non-BRD conditions, such as injury, or were slaughtered as part of normal feedlot management. Euthanasia decisions were made based on veterinary advice in accordance with the Australian Animal Welfare Standards and Guidelines for Cattle. No animals were euthanised specifically for this study.

Cattle were either euthanised for welfare reasons, died naturally or were slaughtered under routine feedlot management in an abattoir. Post-mortem examination was performed immediately after death, euthanasia or slaughter. Gross and histopathological examinations were undertaken for all samples. Lung tissue was aseptically collected, within 1 h of death or euthanasia, from the left lung at the interface between normal and consolidated tissue in BRD cases, or from normal tissue in non-BRD cattle. One portion of the lung tissue was snap-frozen on dry ice and stored at −80 °C until RNA extraction, and a second portion was fixed in 10% neutral buffered formalin for histopathological examination. All 23 BRD cases showed gross and histopathological lesions consistent with respiratory disease, whereas no gross or microscopic lesions were identified in any of the nine non-BRD control cattle, as described in Werid et al. [12]. Study cattle characteristics are provided in Additional file 7.

### RNA extraction and sequencing

Prior to nucleic acid extraction, frozen tissues were thawed on ice and homogenised in sterile phosphate-buffered saline using a bead-beating method. Approximately 30 mg of frozen lung tissue was maintained on ice and transferred to a bead-beating tube containing approximately 300 µL of 0.5 mm zirconium beads and 600 µL Buffer RLT supplemented with β-mercaptoethanol. Samples were homogenised using a TissueLyser II device (Qiagen, Hilden, Germany). Total RNA was then extracted using the RNeasy Mini Kit (Qiagen) according to the manufacturer’s instructions. RNA concentration was measured using the Qubit RNA Broad Range Assay Kit (Thermo Fisher Scientific, Waltham, MA, USA). RNA integrity was assessed using an Agilent TapeStation with RNA ScreenTape (Agilent Technologies, Santa Clara, CA, USA), and samples with RNA integrity number equivalent (RINe) ≥ 7.0 were used for library preparation. Strand-specific libraries were prepared using the Illumina Stranded Total RNA Prep, Ligation with Ribo-Zero Plus Kit with unique dual indexes (Illumina, San Diego, CA, USA), targeting approximately 50.0 million read pairs per sample. Sequencing was performed on an Illumina NovaSeq 6000 using an S4 flow cell in a 2 × 150-bp paired-end configuration, with samples distributed across multiple lanes. Quality control was conducted with FastQC v0.11.9 [18]. Adapter sequences and low-quality bases were removed using Trimmomatic v0.39 [19], using sliding window trimming (4 bp, quality score of 20 [Q20]), leading/trailing base trimming (Q20), and a minimum length threshold of 36 bp. Sample-level quality assessment, based on consistently low inter-sample Pearson correlation, a principal component analysis (PCA) deviation of > 3 standard deviations from the group centroid and lower sequencing depth than the cohort median, identified one BRD sample as an outlier and this sample was excluded from all subsequent analyses, resulting in a final dataset of 31 samples (22 BRD cases and 9 non-BRD cases). For weighted gene co-expression network analysis (WGCNA), an additional BRD sample was removed following standard WGCNA network-based outlier detection where hierarchical clustering identified this sample as a singleton cluster, and its standardised connectivity (Z.k) fell below the threshold of −2.5 [20, 21], yielding 30 samples (21 BRD, 9 non-BRD) for network construction.

Trimmed reads were aligned to the *Bos taurus* reference genome (ARS-UCD1.2) using STAR v2.7.10a [22], with Ensembl v108 annotation. Gene-level read counts for host transcriptome analysis were generated using featureCounts v2.0.3 [23], with paired-end, strand-specific parameters.

### Pathogen detection and abundance estimation

Prior to transcriptome analysis, the same sequencing libraries were previously used for metatranscriptomic profiling of non-host reads to identify and quantify BRD-associated pathogens. Pathogen detection was performed on non-host reads (i.e. reads not mapping to the *Bos taurus* reference genome) using Kraken2 v2.1.2 [24] and then pathogen abundance was estimated using Bracken v2.6.2 [25], as described in Werid et al. [12]. Pathogen abundance was expressed as reads per million non-host reads (RPM).

### Differential expression and functional enrichment analysis

Genes were retained if counts per million (CPM) were ≥ 1.0 in at least three samples. Count data were normalised using the DESeq2 median-of-ratios method (relative log expression [RLE]) and subjected to differential expression analysis using DESeq2 v1.38.3 [26]. Differentially expressed genes (DEGs) between BRD and non-BRD cases were then assessed with genes considered significant at false discovery rate (FDR) < 0.05 and |log_2_ fold change|> 0.585 (corresponding to 1.5-fold change [FC]). Effect sizes were estimated as shrunken log_2_ fold changes using the apeglm method [27].

**Figure 1:**
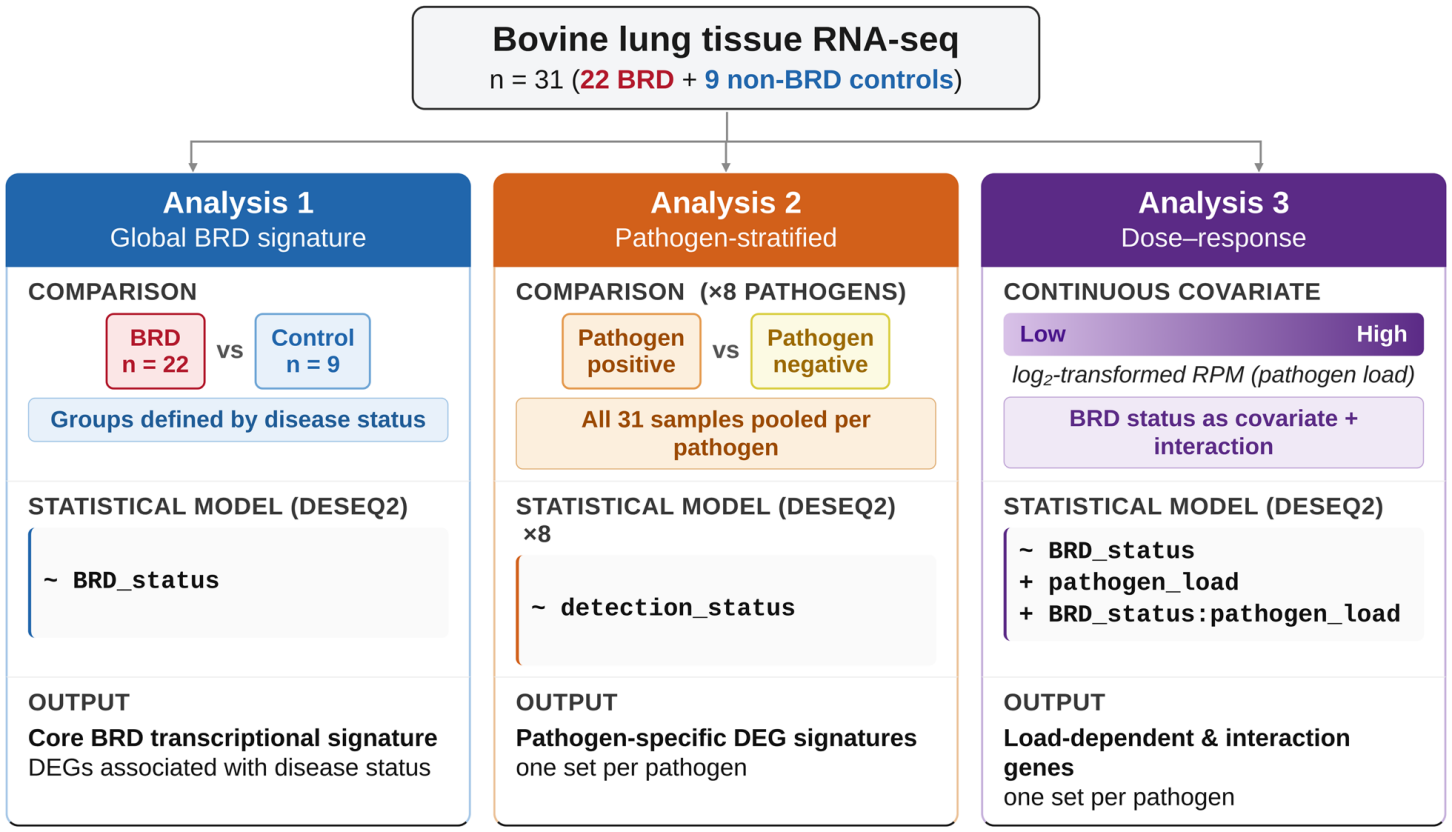
**Differential expression analysis design.** Three complementary RNA-seq analyses were performed on bovine lung tissue from cattle with BRD (*n =* 22) and non-BRD controls (*n =* 9; 31 samples in total after exclusion of one outlier following quality control [QC]). Analysis 1 compared BRD cases with controls to define the overall BRD signature. Analysis 2 compared pathogen-positive with pathogen-negative samples for each of eight pathogens detected in ≥ 3 samples. Analysis 3 modelled as a continuous variable to identify load-responsive host genes, including BRD status and BRD-by-load interaction terms; analyses were restricted to pathogens with coefficient of variation (CV) > 50% in both groups. BRD, Bovine respiratory disease; DEGs, differentially expressed genes; RNA-seq, RNA sequencing; RPM, reads per million non-host reads.

To characterise pathogen-specific host responses, DEG analysis was performed separately for each pathogen, comparing samples in which the pathogen was detected against those in which it was not detected. These comparisons were conducted across the full sample set (i.e. BRD cases and non-BRD controls combined). Pathogen detection status was determined from meta-transcriptomic classification of non-host reads using Kraken2/Bracken. For bacterial pathogens, samples were classified as positive when species-level read counts reached ≥ 25 reads; for viral pathogens, samples were classified as positive when any species-level reads were detected (> 0 reads). Pathogens were considered for inclusion in the analysis based on the findings of Werid et al. [12]. Only pathogens detected in ≥ 3 samples were included to ensure sufficient statistical power for DEG analysis. Eight pathogens satisfied the inclusion criteria for pathogen-stratified analyses: (1) an established aetiological association with the BRD complex; (2) history of detection at the study feedlot through routine surveillance (bacterial culture, polymerase chain reaction (PCR), and 16S ribosomal ribonucleic acid [rRNA] gene sequencing); and (3) detection in ≥ 3 samples in the current dataset. This set of eight pathogens comprised four *Mollicute* species (*Metamycoplasma alkalescens*, *Metamycoplasma canadense*, *Mycoplasmopsis arginini* and *Mycoplasmopsis bovis*), three Gram-negative bacteria (*P. multocida*, *H. somni* and *M. haemolytica*), and one virus (BVDV) (Figure 1). BoHV-1 was detected in only two samples and was excluded from pathogen-stratified analyses.

To assess whether pathogen-associated transcriptional changes were confounded by or independent of BRD status, pathogen contribution to the overall BRD signature was quantified using three complementary methods: overlap percentage (proportion of pathogen DEGs also differentially expressed in the global BRD comparison); directional concordance (proportion of overlapping genes with matching direction of change); and Jaccard similarity index (*J*; intersection divided by union of DEG sets).

Gene Ontology (GO) and KEGG (Kyoto Encyclopedia of Genes and Genomes) pathway enrichment were performed using clusterProfiler v4.6.0 [28]. Hallmark gene set enrichment used fgsea v1.24.0 [29] against MSigDB v7.5 [30]. Gene set variation analysis (GSVA) was used to calculate sample-level enrichment scores using GSVA v1.46.0 [31].

### Cellular deconvolution and dose–response analysis

Bovine Ensembl IDs were mapped to gene symbols using org.Bt.eg.db prior to xCell analysis. Cell-type composition was estimated using xCell v1.1.0 [32], which infers 64 immune and stromal cell type abundances from bulk transcriptomic data. Differences in xCell-inferred cell-type enrichment scores between BRD and control samples were assessed using Wilcoxon rank-sum tests with FDR correction.

Pathogen load was defined as log_2_-transformed RPM non-host reads, a relative measure of pathogen abundance derived from metatranscriptomic classification of non-host reads. Load-dependent gene expression was modelled using DESeq2 with the model: ~BRD_status + pathogen load + BRD status: pathogen load, where pathogen load was log_2_-transformed RPM. Genes with significant simple effects of pathogen load or interaction effects (FDR < 0.05) were classified as dose-responsive. Analyses were performed for pathogens with coefficient of variation (CV) > 50% in both groups.

### Weighted gene co-expression network analysis

Weighted gene co-expression network analysis was performed using WGCNA v1.72–1 [20] on variance-stabilised expression values for genes in the top 20% by variance. A signed network was constructed using soft-thresholding power β = 12 (scale-free topology* R*^2^ > 0.80). The adjacency matrix was transformed into a topological overlap matrix (TOM), and genes were clustered using dynamic tree cutting (minimum module size 20, deepSplit 2, mergeCutHeight 0.25). Module eigengenes were correlated with BRD status and pathogen loads using Pearson correlation with FDR correction. Module preservation was assessed using the modulePreservation function with 200 permutations, calculating Z-summary statistics for each module across subsets excluding each pathogen.

### Host–pathogen protein–protein interaction analysis

Known interactions between BRD pathogen effector proteins and bovine host targets were compiled from literature-curated databases, including the Host–Pathogen Interaction Database (HPIDB 3.0), IntAct and VirHostNet. For pathogens with limited bovine-specific interaction data available, human orthologue interactions were mapped to bovine genes using Ensembl BioMart orthology tables (requiring one-to-one orthology). Interactions were retained if supported by experimental evidence (≥ 2 publications or high-throughput screening). Host target proteins were cross-referenced with the lung transcriptome to determine expression in the tissue of interest.

### Protein–protein interaction network analysis

A protein–protein interaction (PPI) network of BRD-associated DEGs was constructed using STRINGdb v11.5. The *Bos taurus* interactome (species ID 9913) was queried with a combined confidence score threshold ≥ 700. Where bovine interactome coverage was insufficient, human one-to-one orthologues (Ensembl BioMart Compara) were used. Network communities were detected using the Louvain algorithm. Hub genes were ranked by degree and eigenvector centrality.

### Biomarker identification and pathogen ranking

Candidate biomarkers were identified using a multi-evidence scoring approach integrating four components: DEG effect size (40% weight), calculated as |log_2_FC| × −log_10_(FDR); PPI network hub connectivity (25%), based on eigenvector centrality within BRD-associated network communities; dose–response significance (20%), based on the number of significant pathogen load–expression associations; and multi-block integration loading (15%), capturing the contribution to the Data Integration Analysis for Biomarker discovery using Latent cOmponents (DIABLO). Scores were scaled to [0, 1] and summed to produce final biomarker rankings.

Integrated pathogen contribution rankings quantified each pathogen’s transcriptomic impact using four evidence sources: dose–response gene count (30% weight), reflecting direct load–expression associations; PPI network centrality (20%), based on mean eigenvector centrality of pathogen-associated DEGs; transcription factor activity (20%), based on the number of significantly altered transcription factors associated with pathogen presence; and BRD association strength (30%), derived from −log_10_(*p*-value) × log(OR) from a status generalised linear model. All components were scaled to [0, 1] prior to weighted summation.

### Statistical analysis

All analyses were performed in R v4.3.1 [33]. Multiple testing correction used Benjamini–Hochberg FDR. Visualisations were generated using ggplot2 v3.4.2 [34], ComplexHeatmap v2.14.0 [35], and ComplexUpset v1.3.3 [36].

## Results

### Acute-phase and endogenous antimicrobial peptide-associated genes dominate the BRD upregulated signature

Differential expression analysis comparing BRD cases (*n =* 22) with non-BRD controls (*n =* 9) revealed 3148 genes significantly differentially expressed (FDR < 0.05; |log_2_FC|> 0.585), representing 18.7% of the 16,873 genes tested. In addition, differential expression was assessed by comparing pathogen-positive versus pathogen-negative samples for each pathogen across all 31 samples (Additional file 1; Table 1). Upregulation dominated the response: the expression of 2216 genes (70.4%) increased in BRD versus non-BRD cattle, whereas the expression of 932 genes (29.6%) decreased. The magnitude of upregulation generally exceeded that of downregulation, with 235 genes (7.5% of DEGs) showing more than fourfold differences in expression in BRD versus non-BRD cattle. Among the most highly upregulated genes were those encoding acute-phase proteins, including *M-SAA3.2* (log_2_FC = 8.89), *SAA2* (log_2_FC =  8.54), and *SAA3* (log_2_FC = 5.95); antimicrobial proteins, including *SLPI* (log_2_FC = 7.14), *BPIFA1* (log_2_FC = 6.84) and *CATHL2* (log_2_FC = 4.76); chemokines, including *CCL20* (log_2_FC = 6.36), *CXCL2* (log_2_FC = 5.08) and *CXCL8* (log_2_FC = 3.32); and matrix metalloproteinases, including *MMP9* (log_2_FC = 4.64), *MMP3* (log_2_FC = 4.30) and *MMP1* (log_2_FC = 3.73). Key pro-inflammatory mediator genes were also upregulated, including *CSF3* (log_2_FC = 5.32), *TREM1* (log_2_FC = 3.36) and *IL1B* (log_2_FC = 2.83).

**Table 1:** **Pathogen-stratified differential gene expression analysis**

Pathogen	Positive samples (*n*)	Negative samples (*n*)	Upregulated genes (*n*)	Downregulated genes (*n*)	Total DEGs (*n*)
Bovine viral diarrhoea virus	27	4	125	469	594
*Histophilus somni*	25	6	284	641	925
*Mannheimia haemolytica*	28	3	141	80	221
*Metamycoplasma alkalescens*	20	11	523	80	603
*Metamycoplasma canadense*	12	19	763	108	871
*Mycoplasmopsis arginini*	15	16	1571	670	2241
*Mycoplasmopsis bovis*	27	4	267	142	409
*Pasteurella multocida*	28	3	502	715	1217
Total	182	66	4176	2905	7081

Differential expression was assessed by comparing pathogen-positive versus pathogen-negative samples for each pathogen across all 31 samples.

*DEGs* Differentially expressed genes

Downregulated genes showed modest fold changes. The most strongly downregulated genes included those encoding receptors (*VIPR1*, log_2_FC =  −2.35; *SCTR*, log_2_FC =  −1.69), ion channels (*KCNJ11*, log_2_FC =  −1.57), and cell adhesion molecules (*ADAM22*, log_2_FC =  −2.24; *CILP2*, log_2_FC =  −1.65).

### Pathway enrichment confirms coordinated inflammatory signalling and proliferative activation

To interpret these expression changes, we performed gene enrichment analyses. GO enrichment revealed that lungs affected by BRD had a coordinated activation of inflammatory and immune response pathways. The most highly enriched biological processes were “defence response” (84 genes, FDR =  4.79 × 10⁻⁶) and “response to external stimulus” (95 genes, FDR = 5.12 × 10⁻⁶), reflecting a broad immune activation. Cell cycle processes were also over-represented: “mitotic cell cycle process” (68 genes, FDR = 1.20 × 10^−4^) emerged as a significant term, indicating proliferative expansion of infiltrating immune cells. KEGG pathway analysis likewise highlighted interleukin (IL)-17 cytokine signalling (28 genes, FDR =  2.28 × 10^−4^) and cell-cycle regulation (38 genes, FDR = 2.28 × 10^−4^) as key pathways upregulated in cattle with BRD. Several pathways associated with infectious disease and inflammation were also enriched, such as pertussis (FDR =  2.63 × 10^−4^), legionellosis (FDR = 2.64 × 10^−4^) and tumor necrosis factor (TNF) signalling (FDR = 3.13 × 10^−4^), suggesting that host responses to BRD agents in cattle are similar to those that occur in humans for some respiratory pathogen infections.

**Figure 2:**
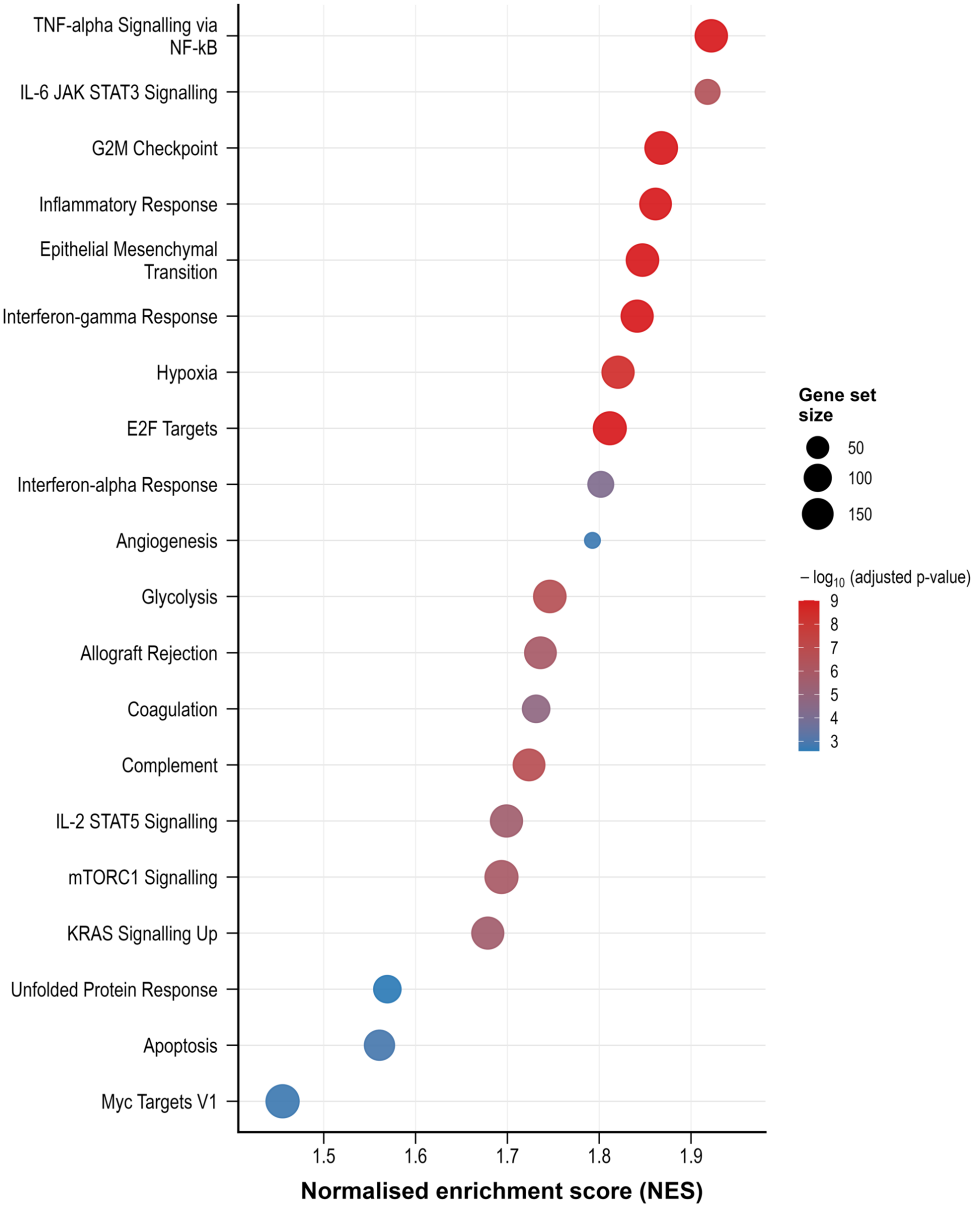
**Hallmark pathway enrichment analysis of transcriptomes from bovine respiratory disease (BRD)-affected lung tissue.** Gene set enrichment analysis showing pathways enriched in lung tissue from cattle with BRD compared with lung tissue from non-BRD controls. The* x*-axis shows the NES. Dot size represents the number of genes contributing to each pathway, and colour intensity represents statistical significance expressed as −log_10_ adjusted *P*-value (*P* < 0.05). Positive NES values indicate pathways enriched in BRD-affected lung tissue. NES, Normalised enrichment score.

Unbiased gene set enrichment analysis (GSEA) confirmed robust activation of hallmark inflammatory pathways in the BRD-affected lungs. Hallmark gene sets for IL-6/JAK/STAT3 signalling, TNF-α signalling via NF-κB, inflammatory response and interferon-γ (IFN-γ) response were all strongly upregulated (normalised enrichment score [NES] 1.88–1.97, FDR on the order of 10^−6^–10^−9^). Concurrently, cell cycle-related gene sets were coordinately enriched, including G2M checkpoint (NES 1.88), E2F targets (1.84) and MYC targets (1.48). Together, these analyses indicate that BRD triggers a dual response of inflammation and cell proliferation. The hallmark of acute BRD is an immune-inflammatory transcriptional response coupled with cell-cycle activation, consistent with lung infiltration by proliferating immune cells (Figure 2).

### Co-expression analysis links BRD to immune activation, cellular proliferation, and structural disruption

**Figure 3:**
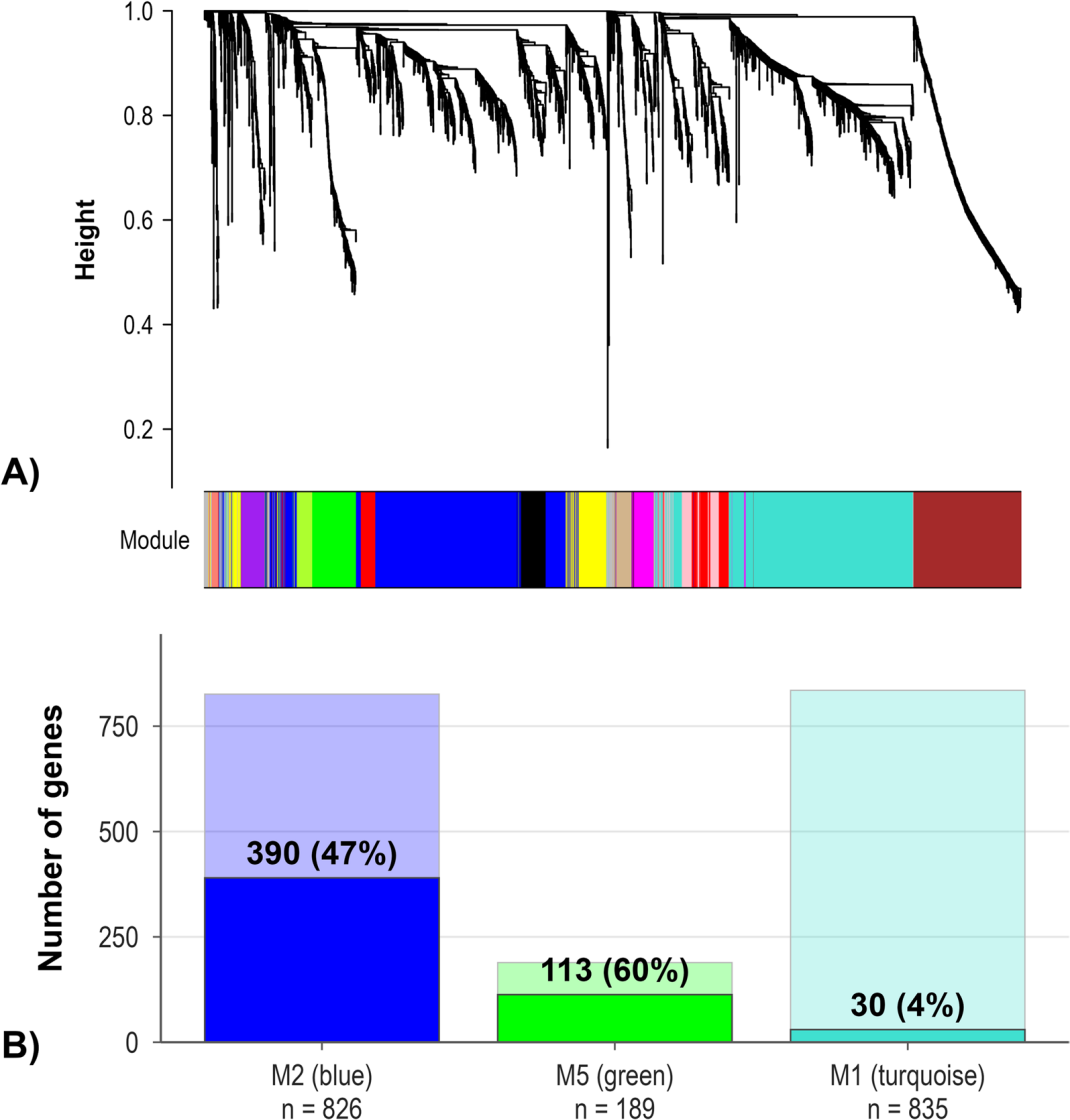
**Weighted gene co-expression network analysis (WGCNA) identifies gene modules associated with bovine respiratory disease (BRD).** WGCNA of the most variable genes in bovine lung tissue. **a** Hierarchical clustering dendrogram of genes based on topological overlap dissimilarity. The colour bar indicates module assignment, with each colour representing a distinct co-expression module. **b** Distribution of BRD-associated differentially expressed genes (DEGs) across BRD-associated WGCNA modules. Bars show the number of DEGs assigned to each module, and percentages indicate the proportion of each module represented by BRD-associated DEGs. WGCNA was conducted using 30 samples after removal of one additional network outlier.

**Figure 4:**
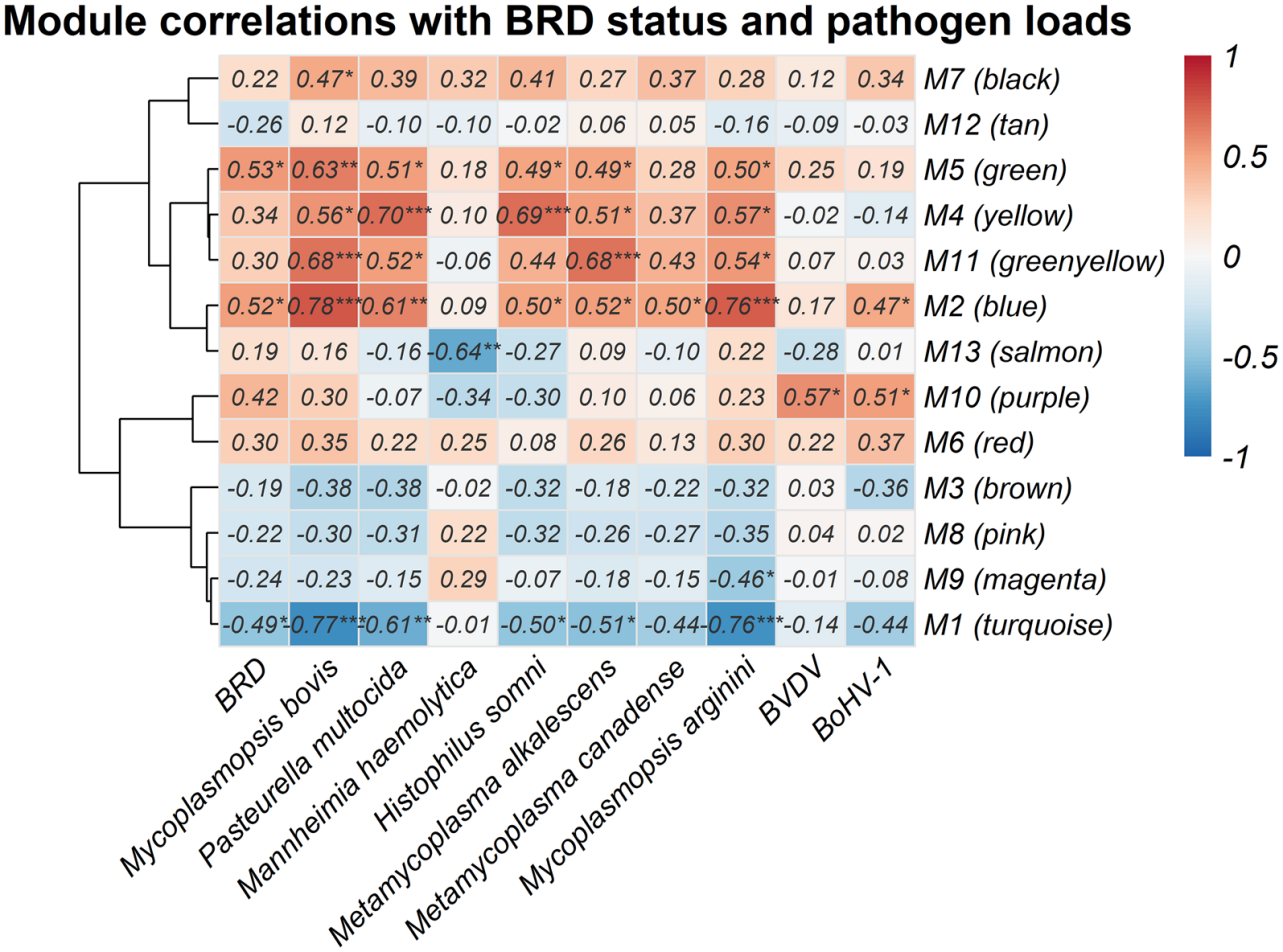
**Module-trait heatmap showing associations between gene co-expression modules and pathogen loads.** Heatmap shows Pearson correlations between weighted gene co-expression network analysis (WGCNA) module eigengenes and sample-level traits, including BRD status and pathogen loads. Rows represent WGCNA modules, and columns represent BRD status or pathogen load. Values in cells of heatmap are Pearson correlation coefficients. Colour coding: red indicates a positive correlation and blue indicates a negative correlation. Asterisks indicate statistically significant associations after correction for false discovery (FDR) (FDR < 0.05). WGCNA was conducted using 30 samples. BRD, Bovine respiratory disease; BVDV, bovine viral diarrhoea virus; BoHV-1, bovine alphaherpesvirus 1;* M*, module.

**Figure 5:**
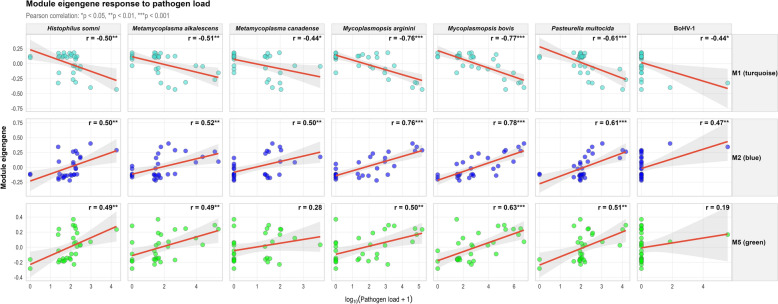
**Module eigengene responses to pathogen load.** Scatter plots showing relationships between selected weighted gene co-expression network analysis (WGCNA) module eigengenes and log-transformed pathogen loads in bovine lung tissue. Each point represents one sample included in the WGCNA dataset. Rows correspond to selected bovine respiratory disease (BRD)-associated modules. Columns represent the pathogen load variables. Fitted lines show linear regression trends, with shaded regions representing 95% confidence intervals. Asterisks indicate statistically significant Pearson correlations (false discovery rate [FDR] < 0.05).

We next applied WGCNA to identify clusters of co-regulated genes underlying the BRD transcriptomic response. This unsupervised approach, applied to the 3375 most variable genes, identified 13 distinct gene modules (Figure 3) ranging in size from 27 to 835 genes. Three of these modules showed a significant association with disease status after adjusting for multiple testing (Figure 4). Specifically, a green module (module 5) and a blue module (module 2) showed strong positive correlation with BRD presence (Pearson *r* ≈ + 0.52, FDR ≈ 0.02), whereas a turquoise module (module 1) was negatively correlated with BRD (*r* = −0.492, FDR = 0.027) (Figure 5).

Each BRD-associated module corresponded to a distinct biological function. The green module (189 genes) was dominated by cell-cycle genes. GO analysis revealed “mitotic cell cycle” as the top enriched term (39 genes, FDR = 1.68 × 10^−34^), with other mitosis-related categories such as “chromosome segregation” (FDR = 4.84 × 10^−23^) also highly significant. Consistently, this module’s gene transcripts were enriched in the KEGG “Cell cycle” pathway (FDR = 4.59 × 10^−16^) and pathway included many core mitotic machinery components; for example, hub genes with the highest connectivity in this module were *KIF11*, *TOP2A*, *NDC80*, *DLGAP5* and *ARHGAP11A*. In contrast, the blue module (826 genes) represented an immune/inflammatory function; its top enriched terms included “chemotaxis” (28 genes, FDR = 8.72 × 10^−13^) and immune pathways like “cytokine–cytokine receptor interaction” (KEGG, FDR = 5.03 × 10⁻^14^). The blue module contained numerous chemokines, cytokine receptors and other immune regulators that orchestrate leukocyte recruitment to the lung. The turquoise module (835 genes) was inversely associated with disease (downregulated in BRD) and was enriched for genes involved in tissue structure and development; for example, it showed strong enrichment for “circulatory system development” (34 genes, FDR = 8.00 × 10^−16^) and “cell adhesion” (FDR = 1.48 × 10^−12^).

We also explored how these gene modules relate to specific pathogens. Module eigengenes (the first principal component [PC] of each module’s expression) were correlated with pathogen loads across samples (Figures 4, 5). This analysis yielded 34 significant module–pathogen associations (FDR < 0.05). The blue immune module strongly correlated with mollicute infection, with its eigengene correlating with *M. bovis* load (*r* = 0.78) and *M. arginini* load (*r* = 0.76) with FDR ≈ 3.7 × 10^−5^ for each. Conversely, the turquoise structural module was negatively correlated with those same pathogens (*r* ≈ −0.77), consistent with *Mollicute*-driven inflammation causing loss of structural gene expression. A yellow module (ME4; 199 genes) enriched for extracellular matrix organisation (FDR = 3.5 × 10^−15^) and proteolysis (FDR = 4.1 × 10^−6^) was strongly induced in samples with high *P. multocida* (*r* = 0.70, FDR = 2.4 × 10^−4^) and *H. somni* (*r* = 0.69, FDR = 3.1 × 10^−4^) loads, consistent with tissue remodelling during bacterial pneumonia. In contrast, a salmon module (ME13; 27 genes) comprising oxidative phosphorylation genes (13/14 KEGG-mapped genes; FDR = 2.0 × 10^−23^) showed a strong negative correlation with *M. haemolytica* load (*r* = −0.64, FDR = 0.002) and detection status (*r* = −0.60, FDR = 0.007). This inverse association was pathogen-specific: the salmon module showed no significant correlation with BRD status overall (*r* = + 0.19, *P* = 0.31), nor with other bacterial pathogens. A similar negative correlation was observed with BVDV status (*r* = −0.55, FDR = 0.026). In summary, the co-expression network analysis indicates that different pathogens preferentially activate different gene consortia in the lungs.

### Inflammation in BRD occurs via shared Mollicutes signatures and dose-dependent Gram-negative effects, whereas bovine viral diarrhoea virus triggers an antagonistic immunosuppressive profile

**Figure 6:**
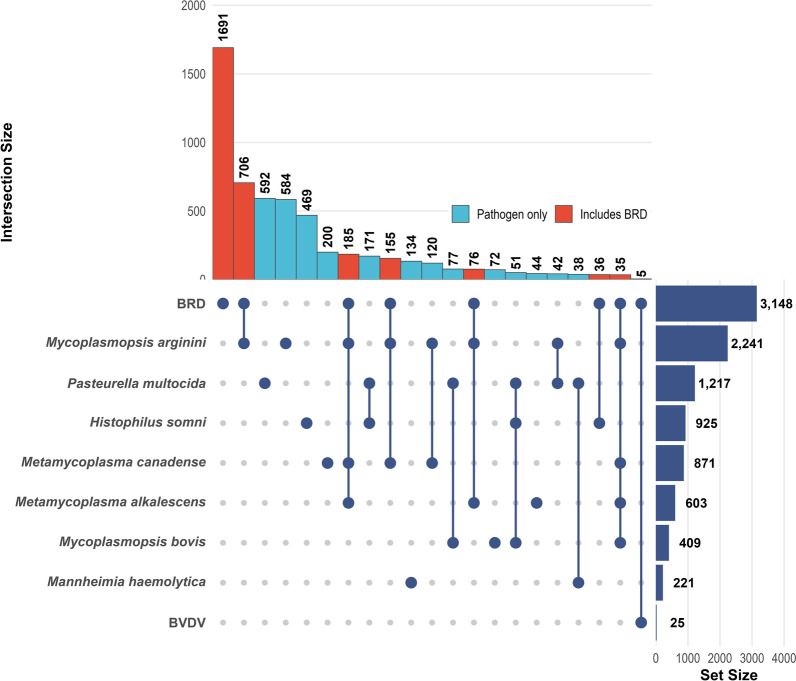
**Unique and shared differentially regulated gene (DEG) intersections in BRD and pathogen-associated comparisons.** The UpSet plot shows unique and shared DEG intersections in BRD and pathogen-associated comparisons. Each set represents genes significantly differentially expressed in the BRD comparison or in a pathogen-positive versus pathogen-negative comparison. Vertical bars show the number of DEGs unique to each displayed intersection, and connected dots identify the DEG sets contributing to that intersection. Set-size bars show the total number of DEGs in each BRD or pathogen-associated comparison. Intersections containing BRD represent genes shared between the core BRD transcriptional response and pathogen-associated responses, whereas pathogen-only intersections represent genes shared among pathogen-associated signatures. DEGs are defined as false discovery rate (FDR) < 0.05, |log_2_ fold change (FC)| > 0.585. BRD. Bovine respiratory disease; BVDV, bovine viral diarrhoea virus.

**Figure 7:**
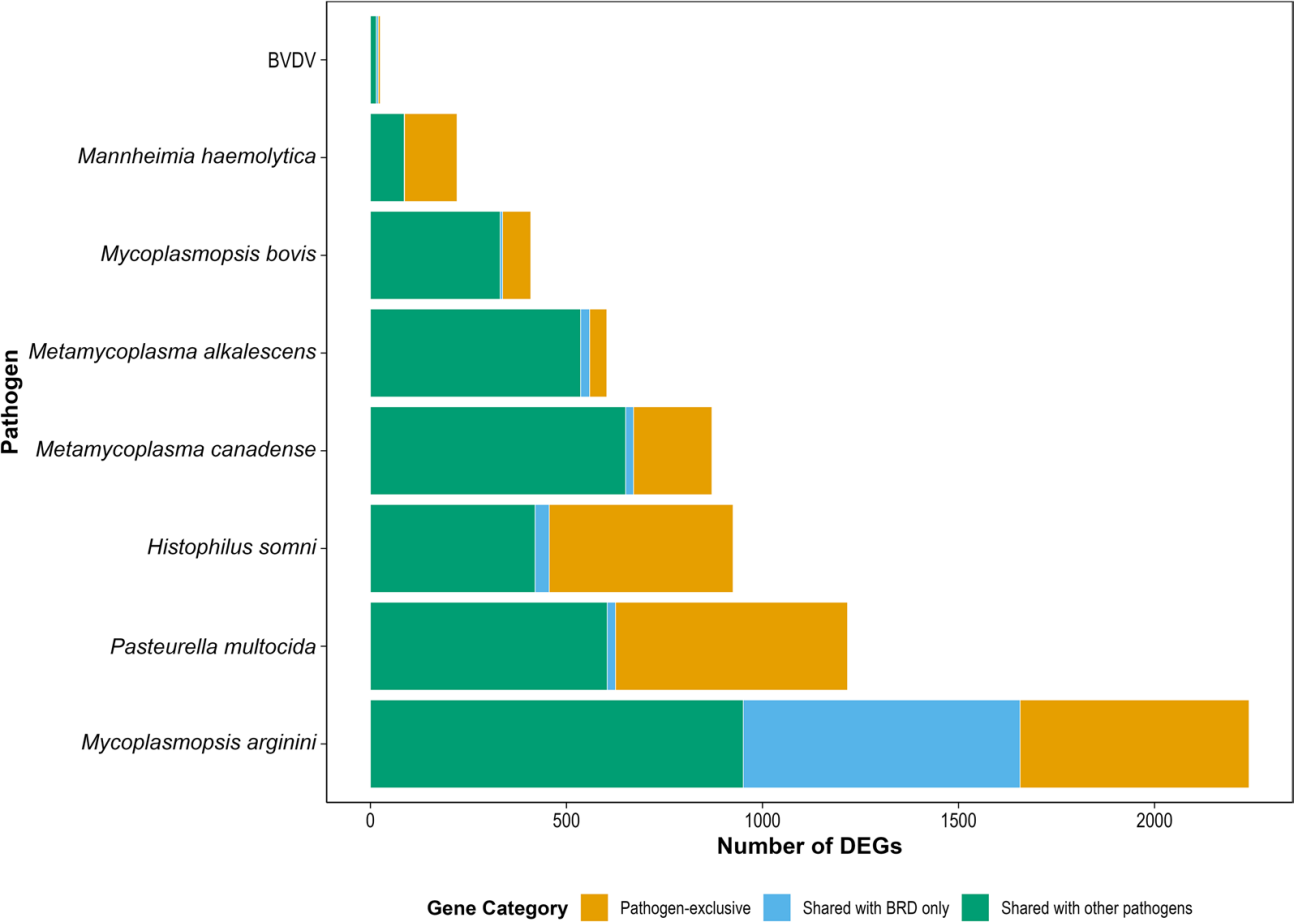
**Exclusivity of pathogen-associated DEG signatures.** Stacked bar plot showing the classification of pathogen-associated DEGs according to whether they were pathogen-specific (orange), shared with the BRD signature only (blue) or shared with other pathogen-associated signatures (green). Each bar represents one pathogen-associated DEG set. Shown in the proportion of each pathogen’s DEGs that were exclusive to that pathogen (orange), shared with the BRD signature only (blue) or shared with other pathogens (green). DEGs were defined as false discovery rate (FDR) < 0.05, |log_2_ fold change (FC)| > 0.585. BRD, Bovine respiratory disease; BVDV, bovine viral diarrhoea virus; DEGs differentially regulated genes.

**Figure 8:**
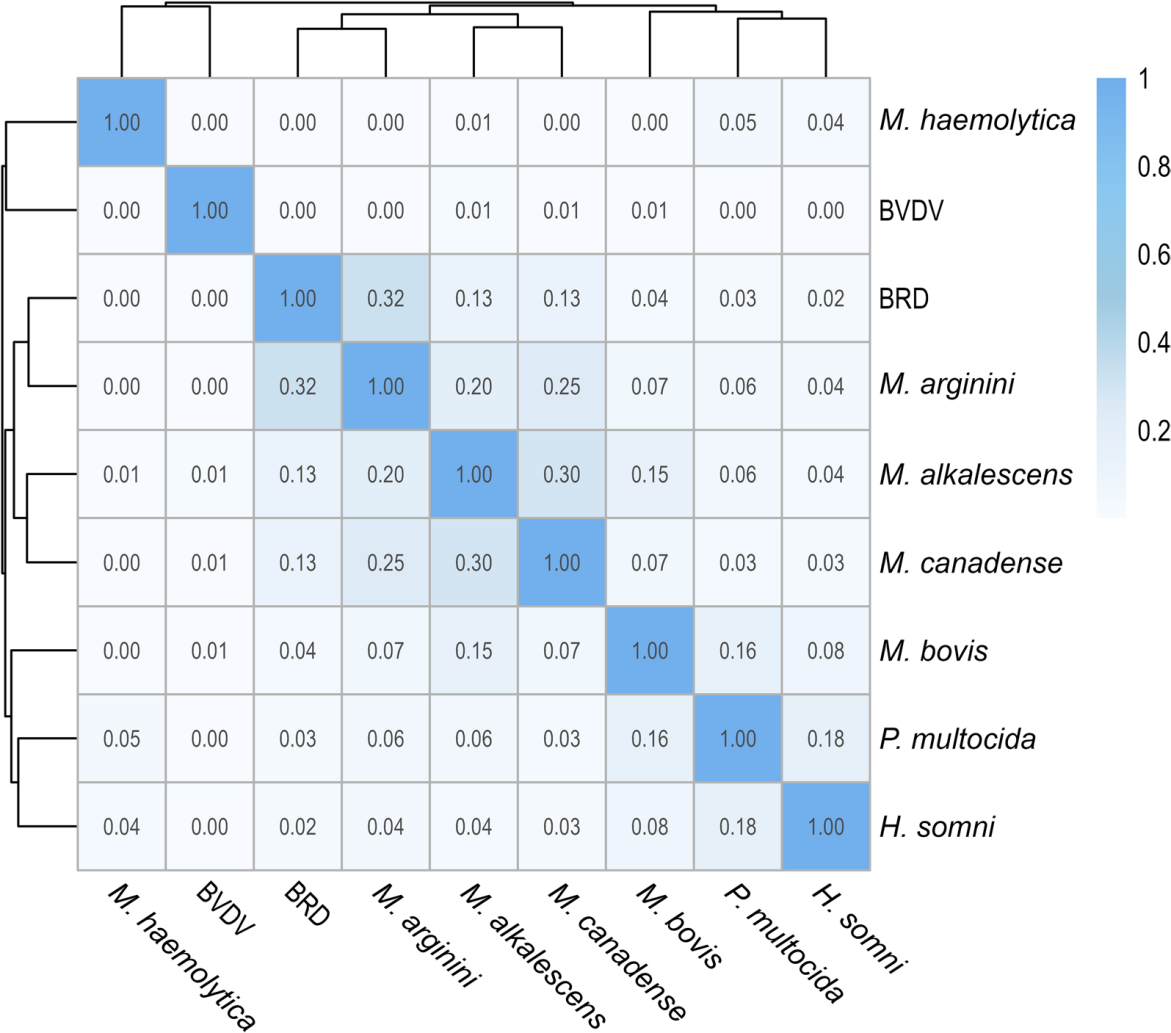
**Pairwise Jaccard similarity among BRD and pathogen-associated differentially regulated gene (DEG) sets.** Heatmap showing pairwise Jaccard similarity indices (*J* )among BRD-associated and pathogen-associated DEG sets. The Jaccard index was calculated as the number of shared DEGs divided by the union of the two DEG sets. Values inside cells of the graph represent pairwise Jaccard similarity. Darker shading indicates greater similarity between DEG sets. Dendrograms show hierarchical clustering of BRD and pathogen-associated transcriptional signatures. Pathogens include *Mycoplasmopsis arginini* (*M. arginini*), *Mycoplasmopsis bovis* (*M. bovis*), *Metamycoplasma alkalescens* (*M. alkalescens*), *Metamycoplasma canadense* (*M. canadense*), *Pasteurella multocida* (*P. multocida*), *Histophilus somni* (*H. somni*), *Mannheimia haemolytica* (*M. haemolytica*), and BVDV. DEGs are defined as false discovery rate (FDR) < 0.05, |log_2_ fold change (FC)| > 0.585. BRD, Bovine respiratory disease; BVDV, bovine viral diarrhoea virus.

**Figure 9:**
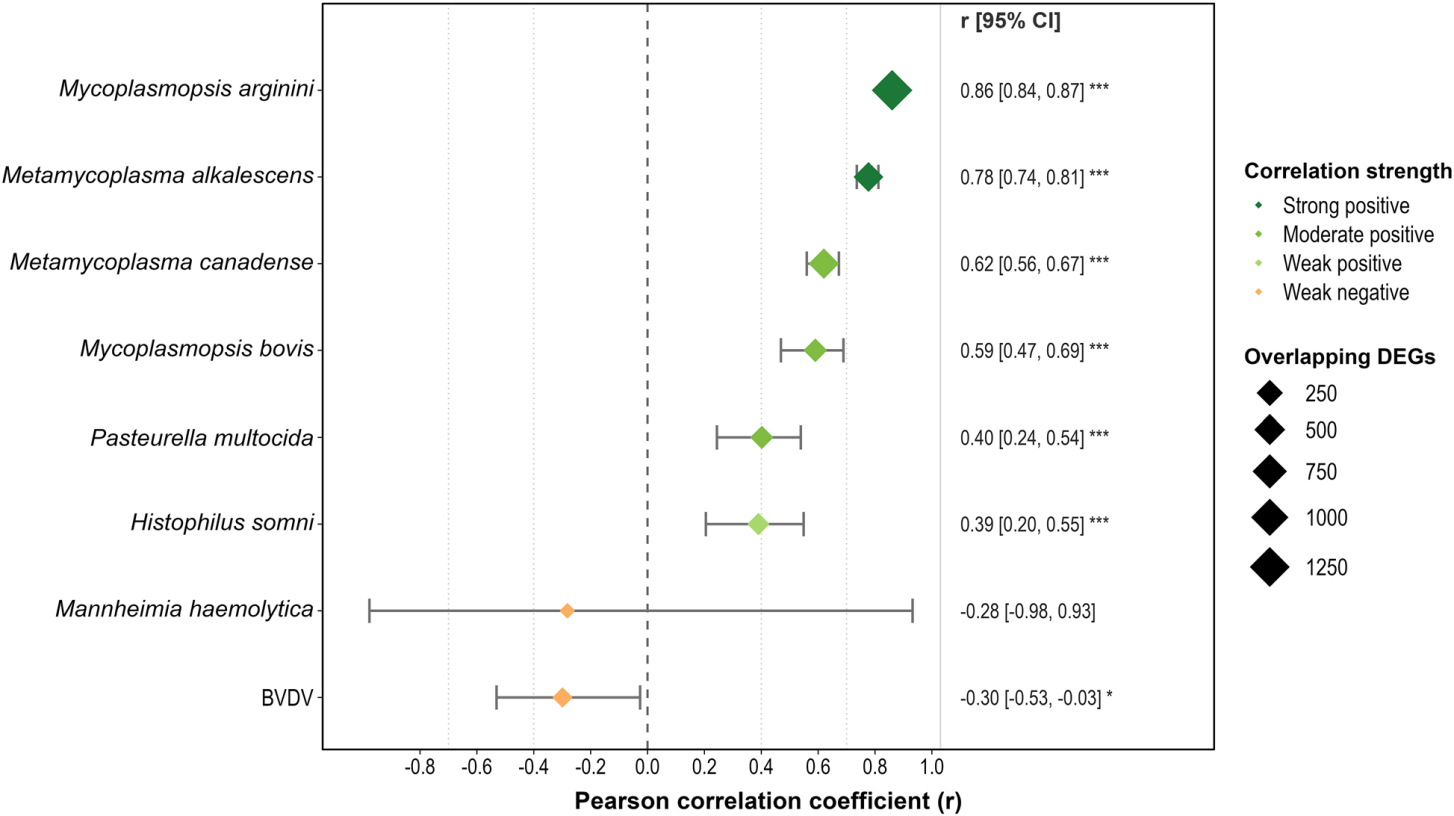
**Transcriptional concordance between pathogen-associated and bovine respiratory disease (BRD)-associated gene expression changes.** Forest plot of Pearson correlation coefficients (*r*) comparing log_2_ fold change (FC) values for DEGs shared between each pathogen and the BRD comparison. Horizontal bars represent the 95% CI; diamond size is proportional to the number of overlapping DEGs. The dashed vertical line indicates *r* = 0, dotted vertical lines indicate *r* = ±0.4 and ±0.7. Colour coding: Green, positive concordance (FC in the same direction); orange–red, negative concordance (FC in opposite directions). Asterisks indicate the statistical significance of the Pearson correlation: *P < 0.05, **P < 0.01, ***P < 0.001. BVDV, Bovine viral diarrhoea virus; CI, confidence interval; DEGs, differentially expressed genes.

Pathogen-specific analysis revealed higher heterogeneity in host transcriptional responses (Figures 6–9; Additional file 2). Despite moderate prevalence (48.4% of samples), *M. arginini* showed the strongest transcriptional association with the BRD inflammatory response among the pathogens examined (2241 DEGs) (Figure 6). Of these, 1316 (58.7%) overlapped with the core BRD signature, accounting for 41.8% of all BRD DEGs and yielding the highest pairwise Jaccard similarity observed (*J* = 0.32; Figures 7, 8). All overlapping genes were regulated in the same direction (100% concordance), with fold changes strongly correlated between *M. arginini* and BRD responses (*r* = 0.86, *P* < 0.0001; Figure 9). Hierarchical clustering of the Jaccard similarity index confirmed that *M. arginini* was transcriptionally most similar to the aggregate BRD signature (Figure 8). Other Mollicutes showed comparable pro-inflammatory profiles. *Metamycoplasma alkalescens* (*J* = 0.13, *r* = 0.78) and *M. canadense* (*J* = 0.13, *r* = 0.62) clustered together and showed strong positive correlations with BRD, while *M. bovis* (*J* = 0.04, *r* = 0.59) showed only moderate concordance. In these species, 65–88% of DEGs were upregulated, reinforcing the association between Mollicutes and the pulmonary inflammatory transcriptome in BRD.

In contrast, BVDV showed a distinct immunosuppressive signature. BVDV-positive samples produced 594 DEGs, of which 79% were downregulated, with minimal transcriptional overlap with BRD (*J* = 0.01; Figure 8). Only 25.5% of the 51 overlapping genes changed concordantly (74.5% discordant), and BVDV fold changes were negatively correlated with BRD (*r* = −0.30, *P* = 0.033; Figure 9).

To quantify how pathogen load influenced gene expression, we performed dose–response analysis. For each pathogen, generalised linear models were used to identify genes whose expression levels scaled with the pathogen’s load in the sample. *Histophilus somni* and *P. multocida* showed the highest load-dependent effects on the host transcriptome (Additional file 3). In *H. somni*-infected lungs, the expression of 64 genes was significantly associated with *H. somni* load. Similarly, *P. multocida* load affected 53 genes. Multiple genes responded to pathogen load only in the context of disease (significant load × BRD interactions), suggesting that heavy bacterial loads amplify gene expression changes particularly in already-diseased lungs. Smaller numbers of dose-responsive genes were detected for *M. bovis* (16 genes), BVDV (13 genes) and *M. alkalescens* (5 genes). By contrast, no significant load-associated genes were found for *M. haemolytica*, *M. arginini* or *M. canadense*, indicating that for these pathogens mere presence/absence was the main determinant of transcriptional impact in this study.

We identified a set of pathogen-specific biomarker candidates to aid in determining the aetiological agent in BRD cases. Specifically, we catalogued genes that were differentially expressed exclusively in association with a single pathogen (and not with any other), reasoning that such genes could serve as signatures of that particular infection. This analysis yielded 2430 pathogen-exclusive marker genes (Additional file 4). The breadth of these unique signatures varied greatly by pathogen. BVDV infection induced the largest exclusive signature, 411 genes, comprising 69.2% of all BVDV-related DEGs, highlighting its highly distinct effect on the host transcriptome. The major bacterial pathogens each had roughly half of their DEGs unique to that pathogen: for example, *H. somni* had 463 exclusive markers (~50% of its DEGs); *M. haemolytica*, 108 (48.9%); and *P. multocida*, 588 (48.3%). In contrast, the Mollicutes shared much of their induced gene profiles with one another, resulting in lower proportions of pathogen-specific markers. For example, *M. arginini* had 544 exclusive markers (24.3% of its DEGs), and *M. alkalescens* had only 44 (7.3%), reflecting an overlap in host response pathways triggered by the Mollicutes group.

### BRD lungs show innate immune cell enrichment and PPI network hubs linking inflammation to oxidative stress

**Figure 10:**
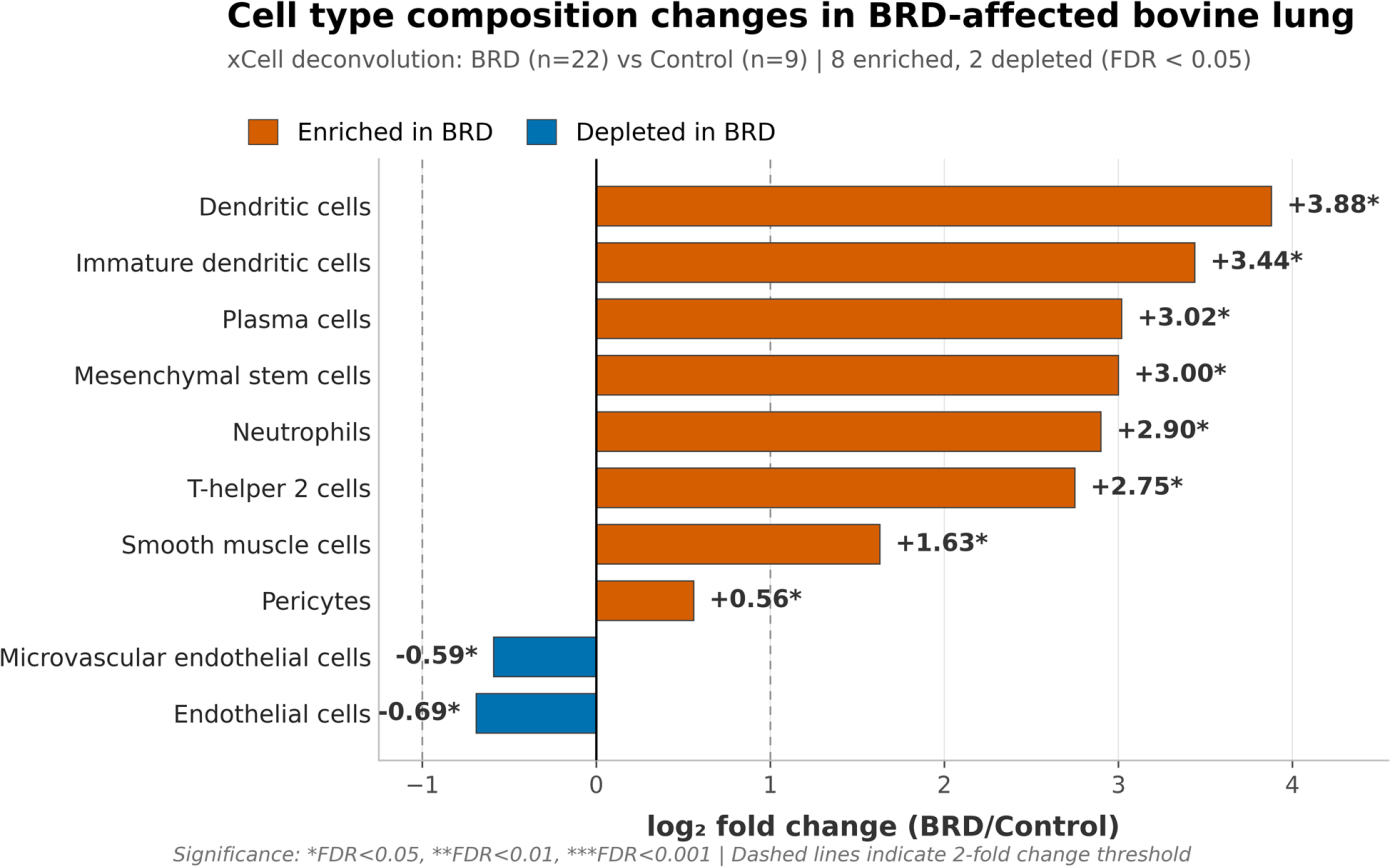
**Inferred cellular composition of BRD-affected bovine lung tissue.** xCell deconvolution of RNA sequencing (RNA-seq) data was used to infer differences in cell-type composition between lung tissue from BRD-affected and non-BRD control cattle. Bars represent log_2_ fold change in xCell enrichment scores for BRD relative to control samples. Colour coding: orange and blue bars indicate cell types enriched and depleted in BRD-affected lung tissue, respectively. Dashed vertical lines indicate two-fold change thresholds. Asterisks denote statistically significant differences after FDR (*FDR < 0.05) correction using Wilcoxon rank-sum tests with Benjamini–Hochberg adjustment. BRD, Bovine respiratory disease; FDR, false discovery rate.

Deconvolution analysis using xCell revealed significant changes in cellular composition within BRD-affected lung tissue (Figure 10). Antigen-presenting cells were significantly enriched: dendritic cells (log_2_FC =  +3.88, FDR = 0.023) and immature dendritic cells (+ 3.44, FDR = 0.023) were elevated 10- to 15-fold relative to controls. Neutrophil infiltration was similarly higher (+2.90, FDR = 0.023), accompanied by expansion of mesenchymal stem cells (+3.00, FDR = 0.023). Evidence of adaptive immune activation included significant enrichment of plasma cells (+3.02, FDR = 0.033) and T-helper 2 cells (+2.75, FDR = 0.027), indicative of active humoral immunity and Th2-polarised inflammation. In contrast, endothelial cells (−0.69, FDR = 0.023) and microvascular endothelial cells (−0.59, FDR = 0.023) were depleted, consistent with inflammation-associated vascular injury.

At the molecular interaction level, we constructed a PPI network from the DEGs. Using the STRING database to connect 462 DEGs with known interactions, we obtained a network comprising > 14 000 protein–protein edges, organised into 11 clusters or communities. The network hubs (genes with highest connectivity) were enriched for inflammatory and metabolic regulators, underscoring their central role in BRD. For example, *SOD2* (superoxide dismutase 2; degree = 184) was one of the top hubs, linking oxidative stress responses to many other proteins. Other high-degree nodes included *MSC* (musculin, degree = 175), *KMO* (kynurenine 3-monooxygenase, degree = 172), *LDHA* (lactate dehydrogenase A, degree = 167) and the chemokine *CXCL2* (degree = 160). These hubs suggest that metabolic reprogramming (e.g. glycolysis and kynurenine pathways), oxidative stress and neutrophil chemotaxis are key interconnected components of the BRD lung response.

To identify molecular points of convergence between the microbes and host immune system, we also examined known host–pathogen protein interactions. Literature curation revealed 48 experimentally verified interactions involving seven BRD-associated pathogens and 35 bovine host proteins (Additional file 5). These interactions highlight how diverse pathogens target common host pathways. For example, *M. haemolytica* secretes leukotoxin (LktA) that binds to β2-integrin receptors (ITGAM/ITGB2) on leukocytes, undermining phagocyte function, and its outer membrane components engage Toll-like receptor 4 (TLR4) and CD14 to activate innate immunity. BVDV possesses Npro and other proteins that suppress IFN-mediated antiviral responses by targeting critical components of the type I IFN pathway (including IFNAR1, STAT1 and STAT2). Other notable interactions include BoHV-1 glycoprotein gB binding to host entry receptors, *M. bovis* variable surface protein A (VspA) interacting with host adhesion molecules, *P. multocida* toxin (PmtA) affecting host cell signalling (dermonecrotic effect), *H. somni* IbpA protein binding immunoglobulins and *M. arginini* ArcA enzyme depleting local L-arginine (a mechanism to evade immune responses). GO analysis of the host proteins targeted by these pathogens showed significant enrichment for immune pathways such as “defence response to symbiont” (6 genes, FDR = 3.35 × 10^−5^) and “type I interferon signalling” (3 genes, FDR = 3.35 × 10^−5^). This indicates that BRD pathogens converge on pivotal host immune signalling nodes, either activating or subverting the same key pathways to influence disease outcome.

## Discussion

We report here the results of a pathogen-stratified transcriptomic analysis of lung tissue from naturally occurring BRD in feedlot cattle. By integrating differential expression, co-expression network analysis, cellular deconvolution and pathogen-stratified approaches, we characterised BRD as a syndrome of inflammatory reprogramming associated with distinct pathogen classes, with each class exhibiting a different transcriptional relationship with the host response. BVDV was associated with an immunosuppressive profile (79% of associated genes downregulated), directionally opposite to the core BRD signature, consistent with the established role of BVDV in impairing host immunity [37–40]. Our data provide field-level evidence of an association with reduced immune gene expression at the time of sampling, although the temporal relationship between BVDV infection and bacterial colonisation cannot be established from this cross-sectional design. Within this immunosuppressed environment, Mollicutes, particularly *M. arginini*, were associated with the characteristic inflammatory transcriptome. *Mollicute*-associated inflammation was strongly associated with colonisation status rather than relative abundance; once established, these organisms might trigger sustained inflammation irrespective of load. In addition, *P. multocida* and *H. somni* were associated with increased tissue damage in a dose-dependent manner, exacerbating the inflammatory cascade. We propose a conceptual model, informed by these associations and prior literature, in which viral immunosuppression is associated with colonisation by Mollicutes and inflammatory gene expression, with Gram-negative bacteria correlating with dose-dependent amplification. This framework provides a basis for understanding the polymicrobial pathogenesis of naturally occurring BRD, although the temporal sequence implied by the model cannot be confirmed from this cross-sectional design.

The magnitude of transcriptional perturbation was striking: 3148 genes (18.7% of the transcriptome) were significantly differentially expressed, with significant bias toward upregulation (70.4%). The most highly induced genes encode acute-phase proteins (*SAA2, SAA3, M-SAA3.2*), antimicrobial proteins (*SLPI*, *BPIFA1*, *CATHL2*) and pro-inflammatory chemokines (*CCL20*, *CXCL2*, *CXCL8*), defining a robust innate immune activation signature consistent with the neutrophilic bronchopneumonia characteristic of BRD [41]. Cellular deconvolution confirmed increased infiltration by dendritic cells, neutrophils and plasma cells. The concurrent enrichment of cell-cycle pathways (G2M checkpoint, E2F targets) likely reflects proliferative expansion of these infiltrating populations rather than epithelial regeneration. The observed transcriptional differences reflect a combination of immune cell infiltration, confirmed by xCell deconvolution, and within-cell transcriptional changes characteristic of activated inflammatory responses, consistent with previous observations in experimentally challenged cattle [15].

Co-expression network analysis using WGCNA [20] revealed that BRD-associated transcriptional changes are organised into functionally coherent modules. Immune-enriched (blue) and proliferative (green) modules were positively correlated with disease status, whereas the structural/extracellular matrix module (turquoise) was negatively correlated, a reciprocal pattern reflecting the displacement of normal alveolar architecture by inflammatory infiltration. This trade-off has direct pathophysiological consequences where the accumulation of immune cells and exudate in alveolar spaces impairs gas exchange, the hallmark of severe pneumonia [42], while breakdown of the extracellular matrix scaffold compromises tissue integrity and repair capacity [43]. Importantly, module eigengenes correlated strongly with pathogen loads, demonstrating that these transcriptional responses are associated with pathogen load rather than merely reflecting binary infection status, an observation that positions module eigengenes as candidate markers of disease activity warranting prospective validation against independent severity endpoints.

The pathogen-stratified analysis revealed an unexpected hierarchy of transcriptional impact among Mollicutes. Although *M. arginini* was detected less frequently (48.4%) than *M. bovis* (87.1%) or other BRD pathogens, the former was associated with substantially more DEGs (2241) than any other pathogen. Moreover, the 1316 genes shared between *M. arginini* and BRD signatures showed complete directional concordance, demonstrating that this mollicute accounts for the core inflammatory programme. The paradox of *M. bovis—*near-ubiquitous detection yet modest transcriptional impact—could be explained by its extensive immune evasion machinery, including variable surface lipoproteins (Vsps) encoded by > 13 genes that undergo high-frequency phase and size variation enabling antigenic switching [44–46], inhibition of lymphocyte proliferation [47], differential modulation of apoptosis (delayed in monocytes, induced in lymphocytes) [48, 49], suppression of IFN-γ and TNF-α production [50], T-cell exhaustion via programmed death-1/programmed death-ligand 1 (PD-1/PD-L1) upregulation [51, 52] and impaired neutrophil respiratory burst [53, 54. These adaptations collectively enable chronic persistent infection with intermittent shedding [55], and our dose–response analysis confirmed few *M. bovis* load-responsive genes, consistent with a host in chronic adapted inflammation where *M. bovis* functions as an effective coloniser but weak inflammatory stimulus [55]. *Mycoplasmopsis arginini*, by contrast, lacks Vsp-like antigenic variation and possesses only 19 of 59* M. bovis* pathogenicity-related genes [56], potentially rendering it more immunologically visible. *Mycoplasmopsis arginini* is not a novel finding in fatal BRD: Gagea et al. [57] reported the isolation of *M. arginini* from 72% of pneumonic lungs in feedlot mortalities, close to that reported for *M. bovis* (82%), yet it has received little attention in subsequent research. Our transcriptomic data now reveal that this historically overlooked pathogen exhibits the strongest association with the BRD inflammatory response. Both species share slow replication kinetics typical of Mollicutes, precluding differential colonisation timing as an explanation; rather, the transcriptional dominance of *M. arginini* likely reflects either unrecognised pro-inflammatory activity, synergistic co-infection interactions or its consistent presence in severe end-stage disease where immune evasion confers no selective advantage. These findings do not diminish the established role of *M. bovis* in BRD but suggest that *M. arginini*, historically detected at comparable prevalence in fatal cases yet largely overlooked [57, 58], may contribute more substantially to the inflammatory pathology than previously recognised. Further longitudinal studies are required to clarify its role relative to *M. bovis*.

The shared inflammatory signatures among Mollicutes species (*M. arginini*, *M. bovis*, *M. alkalescens* and *M. canadense*) suggest common pathogenic mechanisms. Mollicutes induce potent inflammatory responses through membrane lipoproteins that activate Toll-like receptors and through arginine depletion via the arginine deiminase pathway [59, 60]. The *M. arginini* ArcA enzyme depletes local L-arginine, potentially impairing nitric oxide-dependent antimicrobial defences while providing metabolic substrates for bacterial growth [60].

The comparatively attenuated transcriptomic footprint of *M. haemolytica* (221 DEGs, the fewest of any pathogen despite 90.3% prevalence) may reflect vaccine-induced immune priming, as all study cattle had received vaccination against *M. haemolytica*. This contrasts with the larger transcriptomic signatures observed for pathogens not routinely targeted by vaccination, such as *P. multocida* or *H. somni*. However, this interpretation should be made cautiously, as confirmation would require direct comparison with unvaccinated cohorts; alternative explanations, including lower virulence, differences in infection dynamics or competitive displacement by other taxa, cannot be excluded. Human respiratory vaccine studies provide a conceptual parallel, as vaccination can alter pathogen ecology and favour the emergence or expansion of non-target organisms [61, 62]. Such effects are well documented for pneumococcal conjugate vaccines, which have driven serotype replacement [62, 63], and for acellular pertussis vaccines, which have been associated with antigenic divergence in *Bordetella pertussis* [64]. Whether comparable selective pressures operate in BRD remains unknown and will require longitudinal surveillance in vaccinated feedlot populations. Although an inactivated BVDV vaccine (Pestigard®; Zoetis) is registered in Australia, it requires two doses and is not routinely used at feedlot induction [65]. Collectively, the attenuated *M. haemolytica* signature in vaccinated cattle, alongside the prominence of Mollicutes and Gram-negative bacteria, highlights important areas for future research in the Australian feedlot industry.

Beyond vaccine-mediated suppression, *M. haemolytica* showed a unique pathogen-specific signature: the salmon module, comprising mitochondrial oxidative phosphorylation genes, correlated negatively with *M. haemolytica* load (*r* =  −0.64) but showed no association with BRD status overall or with other bacterial pathogens. This pattern is consistent with the cytotoxic activity of leukotoxin A (LktA), a pore-forming repeats-in-toxin (RTX) with high specificity for bovine CD18^+^ leukocytes [66]. Neutrophils and macrophages, the primary LktA targets [66], are the principal infiltrating immune effector cells in BRD; their destruction by leukotoxin would directly reduce the transcriptional signal from these populations, including mitochondrial genes essential for chemotaxis and sustained antimicrobial function [67]. BVDV showed a similar negative correlation with this module (*r* =  −0.55), consistent with its established immunosuppressive effects on leukocyte populations. These findings illustrate how pathogen-specific virulence mechanisms, such as leukotoxin-mediated cytotoxicity for *M. haemolytica* and immune cell suppression for BVDV, generate transcriptional signatures that diverge from the broader inflammatory response associated with *Mollicute*s.

Whereas Mollicutes appear to drive the core inflammatory signature through presence/absence effects, dose–response analyses revealed that *P. multocida* and *H. somni* amplify host inflammation in a load-dependent manner, highlighting the therapeutic implications of the current findings. For Gram-negative pathogens, our data suggest that reducing bacterial load, the primary goal of antimicrobial therapy [68], should attenuate inflammatory gene expression proportionally, consistent with the established efficacy of macrolides and fluoroquinolones (where they are registered) against *Pasteurellaceae* in naturally occurring BRD [69, 70]. However, pathogen load in our study was estimated as RPM, a relative measure of abundance. Compositional changes in polymicrobial samples can alter relative abundance without corresponding changes in absolute microbial load, and this distinction should be considered when interpreting dose–response relationships.

Mollicutes-associated inflammation was linked to colonisation status rather than pathogen relative abundance, implying that once infection is established, further reductions in bacterial load may yield minimal immunological returns. This aligns with clinical observation that *M. bovis* infections are often refractory to antimicrobial treatment despite apparent in vitro susceptibility [71]; conventional *M. bovis* bacterins have shown limited efficacy (≤ 44%) and do not prevent colonisation [72, 73], although a recently commercialised modified-live *M. bovis* vaccine (Protivity®; Zoetis) targeting both cell-mediated and humoral immunity is an important development in the field [74].

BVDV was associated with a different transcriptional signature compared with bacterial BRD pathogens. Mollicutes and Gram-negative bacteria upregulated inflammatory genes, whereas BVDV predominantly downregulated genes (79% of 594 DEGs) and correlated negatively with the BRD signature (*r* =  −0.30; 74.5% directional discordance). This immunosuppressive profile aligns with known BVDV pathobiology where the viral Npro protein disables type I IFN responses by targeting interferon regulatory factor 3 (IRF-3) for proteasomal degradation [37] and sequesters the innate immune effector S100A9 [38], predisposing cattle to secondary bacterial infection [39, 40]. Our transcriptomic data thus provide field-level evidence consistent with the established pathogenic mechanisms of BVDV and reinforce its role as a predisposing agent that impairs host defences rather than directly driving the inflammatory response.

The PPI network analysis revealed that the BRD transcriptional response converges on metabolic and stress-response pathways. *SOD2* emerged as the highest-degree hub, consistent with the enzyme's established role as the primary mitochondrial defence against oxidative stress in the inflamed lung [75]. Other prominent hubs included *KMO* and *LDHA*, implicating tryptophan catabolism and glycolytic reprogramming in BRD pathogenesis. *KMO* occupies a critical branch point in the kynurenine pathway, and its pharmacological inhibition protects against inflammation-induced lung injury in rodent models [76]. *LDHA* catalyses the terminal step of aerobic glycolysis—the metabolic shift characteristic of activated inflammatory macrophages, commonly termed the 'Warburg effect' [77, 78]. The convergence of these pathways suggests that BRD inflammation is accompanied by coordinated metabolic reprogramming, and that these network hubs may represent candidate therapeutic targets or severity biomarkers requiring further investigation.

This study presents a pathogen-stratified transcriptomic analysis of lung tissue from naturally occurring BRD in feedlot cattle. However, several limitations warrant consideration. The cross-sectional design precludes establishing causality or temporal relationships between pathogen colonisation and host responses, and naturally infected samples, while ecologically valid, prevent controlled assessment of individual pathogen contributions. The BRD-affected animals sampled in this study represent chronic, treatment-refractory cases, and the transcriptional profiles may not be generalisable to acute or mild BRD. The modest sample size (*n =* 31) constrained covariate adjustment and precluded cross-validation of the biomarker and pathogen ranking scores, which were assigned a priori from domain knowledge; these rankings are hypothesis-generating and require validation in independent cohorts. Finally, RNA-seq captures transcript abundance but not protein levels or post-translational modifications that may modulate inflammatory responses.

In conclusion, this pathogen-stratified transcriptomic analysis of bovine lung tissue samples revealed a hierarchical pathogen–host interaction where BVDV was associated with suppressed host immunity; Mollicutes, particularly *M. arginini*, showed the strongest transcriptional association with the core inflammatory response; and Gram-negative bacteria correlated with increased inflammation proportionally to microbial load. These findings support BVDV vaccination to preserve immune competence and the consideration of *M. arginini* as a bona fide BRD pathogen alongside *M. bovis*, *H. somni* and *P. multocida*.

## Supplementary Information

**Additional file 1.** **Detection rate of bovine respiratory disease pathogens included in the study.**

**Additional file 2.** **Pathogen-BRD correlation based on differential gene expression analysis.**

**Additional file 3.** **Summary of pathogen dose–response analysis results with model coefficients.**

**Additional file 4.** **List of pathogen exclusive biomarker genes.**

**Additional file 5.** **List of annotated pathogen proteins used for host pathogen interaction.**

**Additional file 6.** **List of accession numbers of RNA seq datasets submitted to NCBI under BioProject PRJNA1340967.**

**Additional file 7.** **Study cattle characteristics including days on feed, antimicrobial treatment history, and clinical classification.**

## Data Availability

Raw RNA-seq data have been deposited in the NCBI Sequence Read Archive under BioProject PRJNA1340967 (individual sample accessions listed in Additional file 6). The datasets supporting the conclusions of this article are included within the article and its additional files.
